# An equivalent perfectly plastic model for estimating squeezing deformations in tunnelling through strain-softening ground

**DOI:** 10.1038/s41598-026-63765-9

**Published:** 2026-08-10

**Authors:** Maria Anthi, Georgios Anagnostou

**Affiliations:** ETH Zurich, Zurich, Switzerland

**Keywords:** Strain-softening materials, Non-localised response, Perfectly plastic model, Peak strength, Residual strength, Engineering, Materials science

## Abstract

A salient feature of stress analysis of strain-softening materials is the loss of solution uniqueness, *i.e*., the existence of several solutions with localisation of plastic deformations in thin shear bands in addition to a response with plastic deformations smoothly distributed over an extended zone. Although the non-localised response is only one among several possible solutions and may underestimate deformations to some extent, it remains of practical and scientific interest because it provides an indication of deformation magnitude and constitutes the basis of numerous analytical and semi-analytical solutions available in the literature. This paper investigates the adequacy of using the widely adopted linearly elastic, perfectly plastic constitutive model in combination with an appropriate strength value (hereafter referred to as “equivalent strength”) to approximate the non-localised response of strain-softening materials to deep tunnel excavation. The equivalent strength lies between the residual strength and the peak strength and is determined such that the perfectly plastic model reproduces the tunnel convergence predicted by the non-localised solution of the classical rotationally symmetric plane-strain problem of an unsupported circular tunnel in softening ground. The proposed approach is subsequently evaluated through numerical analyses of non-rotationally symmetric problems involving non-circular tunnel geometries and anisotropic *in-situ* stress fields. The results show that the equivalent perfectly plastic model generally provides a good approximation of the magnitude and distribution of tunnel convergence predicted by the non-localised strain-softening response across different geometries and stress conditions. The proposed method offers a computationally efficient and numerically robust means for preliminary tunnel design and for estimating the non-localised response of strain-softening ground using constitutive models and analysis tools widely employed in engineering practice.

## Introduction

For continuum mechanics problems involving strain-softening materials, the loss of uniqueness leads to the existence of several admissible solutions satisfying the boundary conditions and the governing equations. In addition to responses where the plastic deformations are localised in thin shear bands, solutions also exist in which the plastic deformations are smoothly distributed over an extended zone (hereafter referred to as “non-localised response”). Although the non-localised response is only one among several possible solutions and may underestimate the deformations to some extent^27^, it remains of practical and scientific interest. From a practical standpoint, it provides an indication of the deformation magnitude and forms the basis of numerous analytical and semi-analytical methods used in tunnel engineering. Furthermore, explicit modelling of localisation generally requires constitutive assumptions involving internal length scales and may lead to results that depend on the adopted regularisation framework. The non-localised response therefore continues to represent a meaningful and widely studied reference solution. Its relevance is evident from the extensive literature on tunnel excavation in strain-softening geomaterials (see, *e.g*^1, 3, 6, 7, 11, 12, 15, 17, 30–33^). However, the analytical or semi-analytical solutions presented in these works are generally restricted to rotationally symmetric problems involving cylindrical tunnels or spherical cavities under uniform and hydrostatic *in-situ* stress conditions. Arbitrary geometries and initial conditions require numerical analyses. In engineering practice, such analyses are commonly performed using linearly elastic, perfectly plastic constitutive models with Mohr–Coulomb or Hoek–Brown yield criteria. These models are widely available in commercial software packages, require relatively few material parameters, and benefit from extensive practical experience and established design methodologies. This paper investigates whether the perfectly plastic model (in combination with an appropriate strength value, hereafter referred to as “equivalent strength *f*_*c*_^*eq*^”) can provide a practical approximation of the non-localised response of strain-softening ground. Such an approach would offer several advantages. Besides avoiding the mesh dependence often associated with the conventional strain-softening simulations widely used in engineering practice, it would allow the use of existing analysis and design tools (*e.g*^4, 16, 19–21, 25, 26^), developed on the assumption of perfectly plastic material behaviour.

The focus is on the convergence of an unsupported tunnel in squeezing ground, a variable of direct practical relevance for applications such as the design of yielding support systems in conventional tunnelling and the assessment of shield jamming risk in mechanised tunnelling.

The equivalent strength could correspond to the residual strength, the peak strength, or some intermediate value. Previous studies^5, 22, 23^ demonstrated that adopting the residual strength may constitute a reasonably conservative simplification in specific cases. However, the residual strength may in other situations prove overly conservative, while adopting peak strength may be unsafe. A rational alternative is therefore to determine an equivalent strength that reproduces a selected benchmark solution of the strain-softening problem. The benchmark considered here is the classical rotationally symmetric non-localised solution for the plane-strain excavation of an unsupported cylindrical tunnel in a homogeneous, isotropic material under uniform hydrostatic *in-situ* stress conditions.

The resulting equivalent strength is subsequently evaluated for problem layouts that do not satisfy this assumption, including non-circular tunnel geometries and anisotropic in-situ stress fields.

The present approach bears some similarity to the investigations of Varas et al^27^., which examined the adequacy of self-similar solutions for approximating the actual behaviour. Both approaches address, directly or indirectly, the non-localised solution of the classical circular tunnel problem. The present contribution differs in that it determines an equivalent strength for use with standard perfectly plastic constitutive models and evaluates the resulting approximation over a broad range of material parameters, including more pronounced softening behaviour, and tunnels with a non-circular cross-section.

The paper is structured as follows: Sect. 2 details the strain-softening constitutive model used. Section 3 describes the determination of the equivalent strength based on the benchmark problem, presenting results via nomograms and an analytical approximation. Section 4 validates the performance of the proposed equivalent perfectly plastic model in various non-rotationally symmetric layouts using FE analysis.

## Strain-softening model

A simple strain-softening constitutive model is adopted. The material behaviour is assumed to be linear elastic according to Hooke’s law and follows the Mohr–Coulomb yield criterion with a non-associated flow rule. Softening is modelled as a decrease in the uniaxial compressive strength *f*_*c*_, while other parameters—friction angle *φ*, dilatancy angle *ψ*, Young’s modulus *E* and Poisson’s ratio *ν*—are assumed constant. The decrease in strength is controlled by the deviatoric plastic strain *γ* ^*pl*^, defined as the difference between the maximum and the minimum principal plastic strain, following an exponential decay function^10^:

1 $$f_{c} = f_{c}^{r} + \left( {f_{c}^{p} - f_{c}^{r} } \right)e^{{ - 3\frac{{\gamma^{pl} }}{{\gamma_{95}^{pl} }}}}$$

where *f*_*c*_ ^*p*^ and *f*_*c*_ ^*r*^ denote the peak and residual uniaxial compressive strengths, respectively, and *γ* ^*pl*^_*95*_ is the deviatoric plastic strain required to cause 95% of the total strength drop (*i.e*., a reduction of 0.95 (*f*_*c*_ ^*p*^—*f*_*c*_ ^*r*^)). While the exponential decay is adopted here, the proposed approach is applicable to other softening laws as well (*e.g.*, linear decay).

### Determination of equivalent strength

As discussed in the Introduction, the present work addresses the non-localised response of strain-softening ground. Accordingly, the benchmark considered in this section is the classical non-localised solution for the rotationally symmetric plane-strain problem of an unsupported circular tunnel of radius *α* in an infinite, homogeneous, isotropic medium subjected to a uniform hydrostatic *in-situ* stress *σ*_*0*_. The rotationally symmetric benchmark is adopted solely for determining the equivalent strength; the adequacy of the resulting approximation is subsequently examined for problem layouts that do not satisfy rotational symmetry.

For the strain-softening material, the rotationally symmetric radial displacement at the tunnel wall* u*_*a,0*,_ is computed using the semi-analytical finite difference method proposed by Lee and Pietruszczak^15^. This displacement can be expressed as a function of the governing dimensionless parameters:

2 $$\frac{{u_{a,0} E}}{{a\sigma_{0} }} = f_{1} \left( {\frac{{f_{c}^{r} }}{{\sigma_{0} }},\,\,\frac{{f_{c}^{p} }}{{f_{c}^{r} }},\,\,\frac{{\gamma_{95}^{pl} E}}{{\sigma_{0} }},\,\,\varphi ,\,\,\psi ,\,\,\nu } \right)$$

The dimensionless term *γ* ^*pl*^_*95*_ *Ε*/*σ*_*0*_ represents the rate of softening relative to the elastic stiffness and the confining stress. A smaller value indicates more brittle behaviour, *i.e.*, rapid strength loss with increasing plastic deformations.

For the perfectly plastic Mohr -Coulomb material with an equivalent strength *f*_*c*_^*eq*^ (and the same *E*, *ν*, *ϕ* and *ψ* as the softening material), the corresponding radial displacement at the tunnel wall is calculated analytically after Anagnostou and Kovári^2^:

3 $$\frac{{u_{a,0} E}}{{a\sigma_{0} }} = f_{2} \left( {\frac{{f_{c}^{eq} }}{{\sigma_{0} }},\,\,\varphi ,\,\,\psi ,\,\,\nu } \right)$$

The equivalent strength *f*_*c*_^*eq*^ is determined by equating the right-hand sides of Eqs. 2 and 3. It is convenient to express the equivalent strength relative to the peak and residual strengths, by introducing a “softening factor” *λ:*

4 $$f_{c}^{eq} = f_{c}^{r} + \lambda \left( {f_{c}^{p} - f_{c}^{r} } \right)$$

where *λ* is between 0 (*f*_*c*_^*eq*^ = *f*_*c*_^*r*^) and 1 (*f*_*c*_^*eq*^ = *f*_*c*_^*p*^). This factor depends on the dimensionless parameters governing the softening behaviour:

5 $$\lambda = f_{3} \left( {\frac{{f_{c}^{r} }}{{\sigma_{0} }},\,\,\frac{{f_{c}^{p} }}{{f_{c}^{r} }},\,\,\frac{{\gamma_{95}^{pl} E}}{{\sigma_{0} }},\,\,\varphi ,\,\,\psi ,\,\,\nu } \right) \in \left[ {0,\,\,1} \right]$$

The softening factor *λ* was quantified for a broad range of the governing parameters: strength ratio *f*_*c*_ ^*p*^/*f*_*c*_ ^*r*^ = 1.5, 2, 3, 4 and 5; normalised residual strength *f*_*c*_ ^*r*^/*σ*_*0*_ = 0.1, 0.2 and 0.3; friction angle *φ* = 20º, 25º and 30º; dilatancy angle *ψ* = *φ*—20º; and Poisson's ratio *ν* = 0.3. These parameter ranges are consistent with values reported in the literature for strain-softening geomaterials. Peak-to-residual strength ratios between 1.5 and 5 have been experimentally observed in soils and rocks, where the residual strength can fall to 20—60% of the peak^13, 18^, while strength to *in-situ* stress ratios of 0.1—0.3 are typical for tunnelling through squeezing rocks^14, 24^. Friction angles of 20º to 30º are characteristic for weak rocks^29^, and the assumption* ψ* = *φ*—20º is common^28^.

**Fig. 1:**
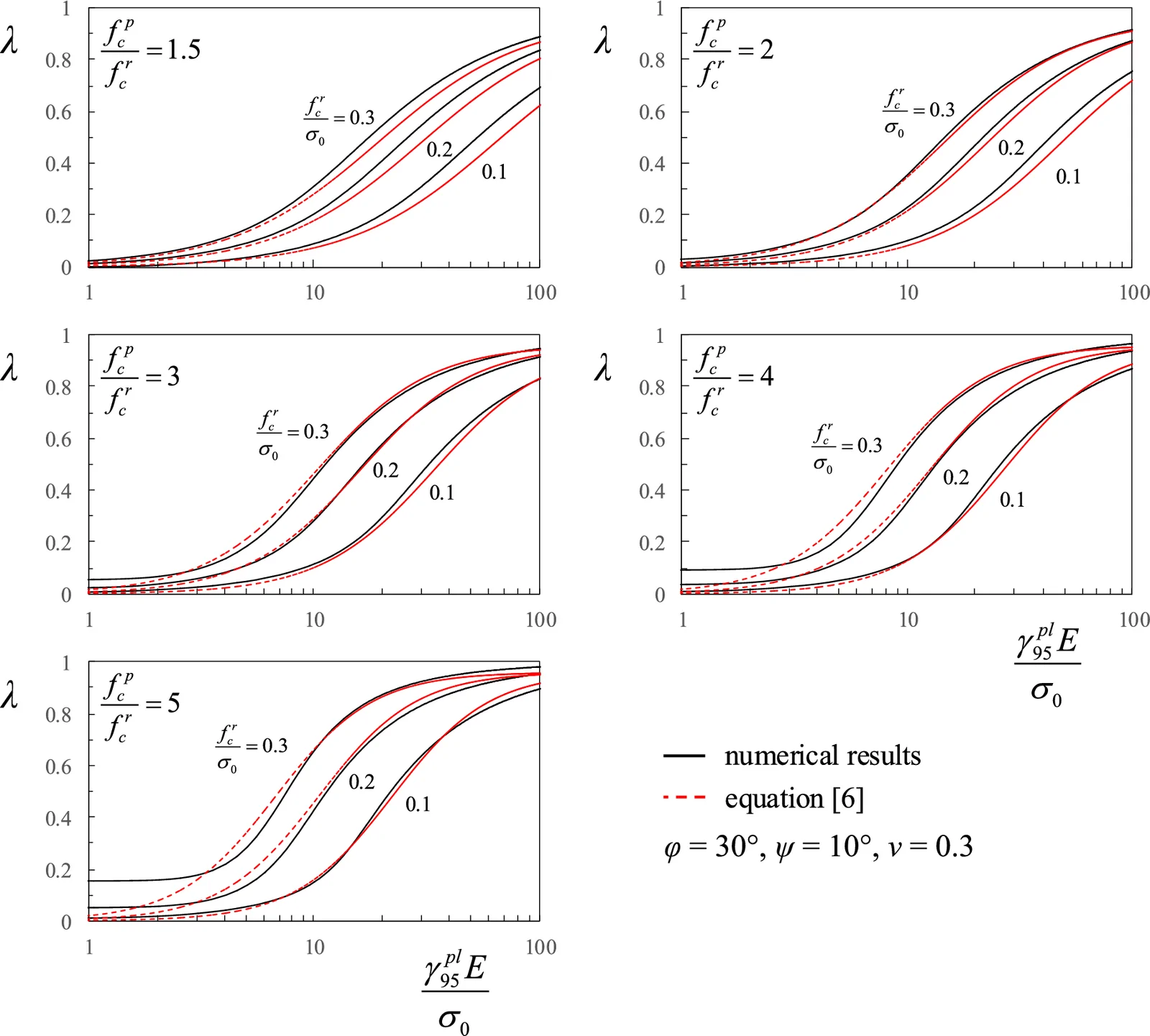
Softening factor *λ* with respect to *γ*^*pl*^_*95*_ *Ε*/*σ*_*0*_, for various *f*_*c*_ ^*p*^/*f*_*c*_ ^*r*^ and *f*_*c*_ ^*r*^/*σ*_*0*_ and fixed parameters *φ* = 30º, *ψ* = 10º and *v* = 0.3.(Black lines show numerical results; red lines show the approximation from Eq. 6.).

**Fig. 2:**
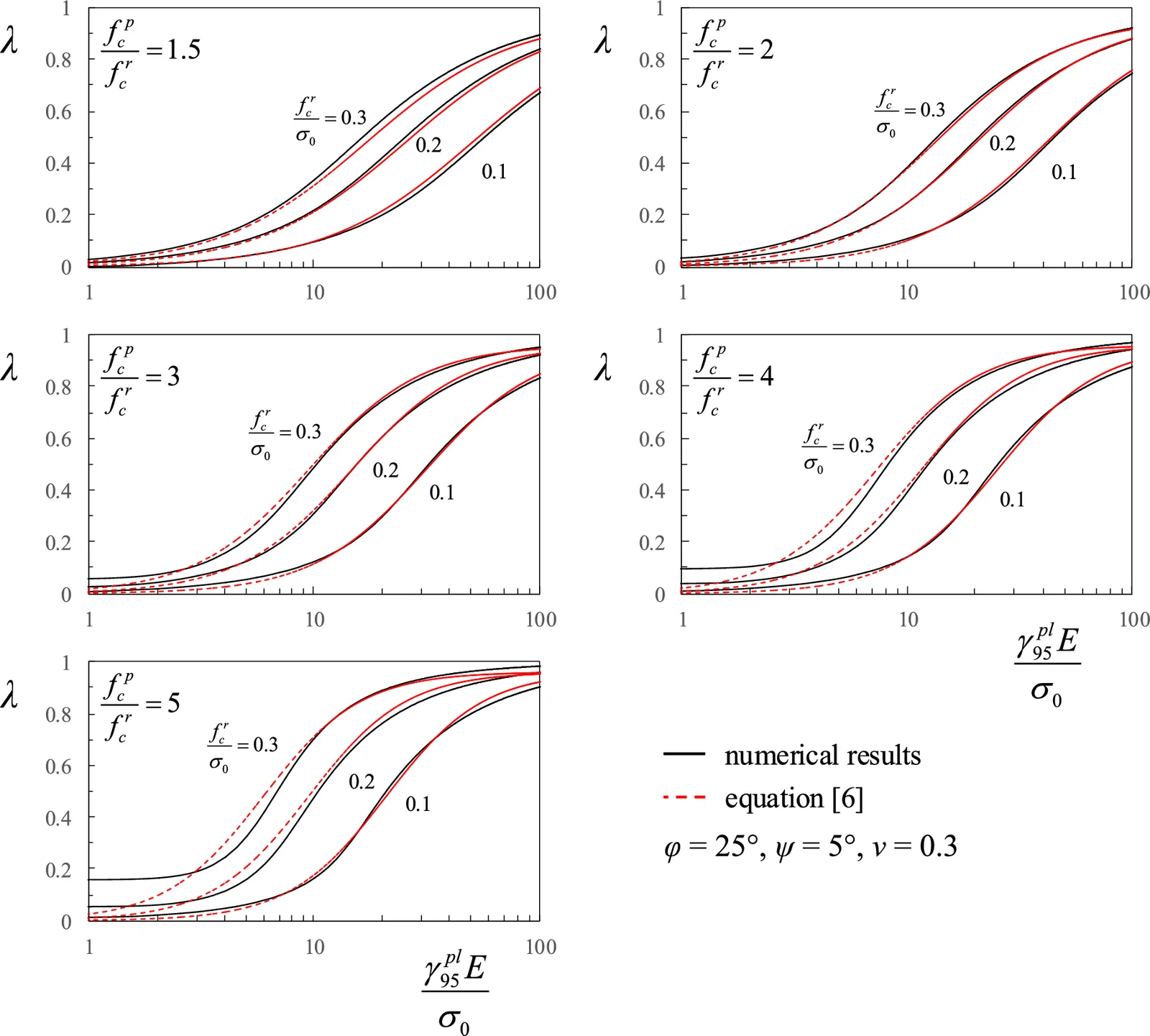
Softening factor *λ* with respect to *γ*^*pl*^_*95*_ *Ε*/*σ*_*0*_, for various *f*_*c*_ ^*p*^/*f*_*c*_ ^*r*^ and *f*_*c*_ ^*r*^/*σ*_*0*_ and fixed parameters *φ* = 25º, *ψ* = 5º and *v* = 0.3.(Black lines show numerical results; red lines show the approximation from Eq. 6.).

**Fig. 3:**
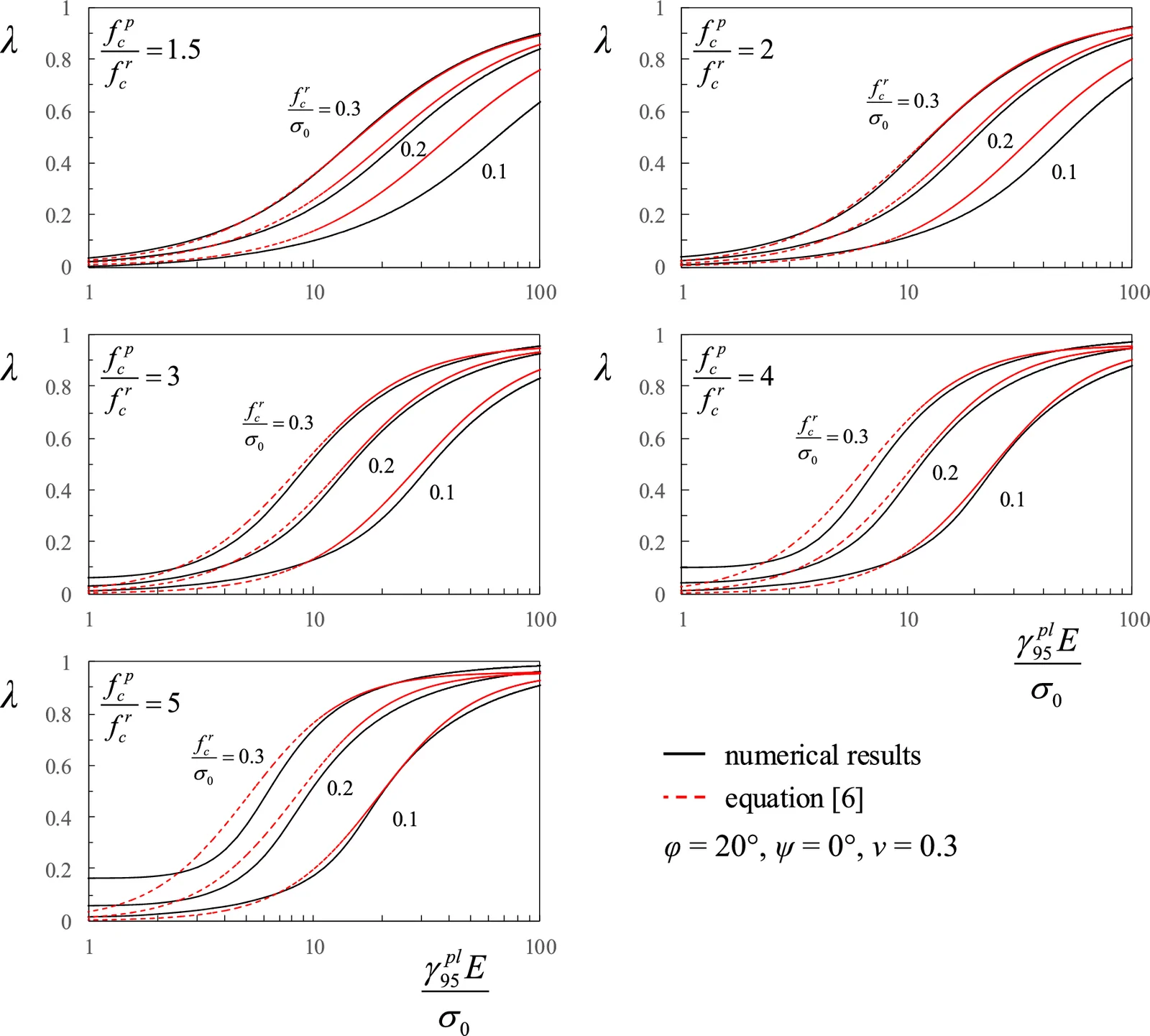
Softening factor *λ* with respect to *γ*^*pl*^_*95*_ *Ε*/*σ*_*0*_, for various *f*_*c*_ ^*p*^/*f*_*c*_ ^*r*^ and *f*_*c*_ ^*r*^/*σ*_*0*_ and fixed parameters *φ* = 20º, *ψ* = 0º and *v* = 0.3.(Black lines show numerical results; red lines show the approximation from Eq. 6.).

The results for φ = 30, 25 and 20° are given as dimensionless *λ* versus *γ*^*pl*^_*95*_*Ε*/*σ*_*0*_ nomograms in Figs. 1, 2 and 3 (black lines), respectively, for all combinations of *f*_*c*_^*p*^/*f*_*c*_^*r*^ and *f*_*c*_^*r*^/*σ*_*0*_. These nomograms, together with Eq. 4, allow the determination of *f*_*c*_^*eq*^ for a given set of material constants and *in-situ* stress. As expected, the equivalent strength, and thus *λ*, increases with increasing *γ*^*pl*^_*95*_*Ε*/*σ*_*0*_, practically from the residual strength (*f*_*c*_^*r*^, *λ* → 0) for very brittle behaviour (small *γ*^*pl*^_*95*_*Ε*/*σ*_*0*_), tending asymptotically to the peak strength (*f*_*c*_^*p*^, *λ*$\to \infty$) for very large *γ*^*pl*^_*95*_*Ε*/*σ*_*0*_ (*i.e*., negligible softening within the relevant strain range, approaching perfectly plastic behaviour at peak strength).

For practical applications, the following analytical approximation for *λ* was developed based on non-linear regression analysis of the computed results:

6 $$\lambda = \frac{0.96}{{1 + a_{1} e^{{a_{2} \frac{{f_{c}^{p} }}{{f_{c}^{r} }}}} \left( {\frac{{f_{c}^{r} }}{{\sigma_{0} }}} \right)^{{\alpha_{3} \ln \left( {\frac{{f_{c}^{p} }}{{f_{c}^{r} }}} \right) + a_{4} }} \left( {\frac{{\gamma_{95}^{pl} E}}{{\sigma_{0} }}} \right)^{{ - 0.56\ln \left( {\frac{{f_{c}^{p} }}{{f_{c}^{r} }}} \right) - 1.12}} }}$$

where

7 $$a_{1} = 6.2\left( {\sin \varphi } \right)^{ - 1.17}$$

8 $$a_{2} = 0.7\ln \left( {\sin \varphi } \right) + 0.2$$

9 $$a_{3} = 1.3\ln \left( {\sin \varphi } \right) + 0.3$$

and

10 $$a_{4} = - 1.4\ln \left( {\sin \varphi } \right) - 2.2$$

**Fig. 4:**
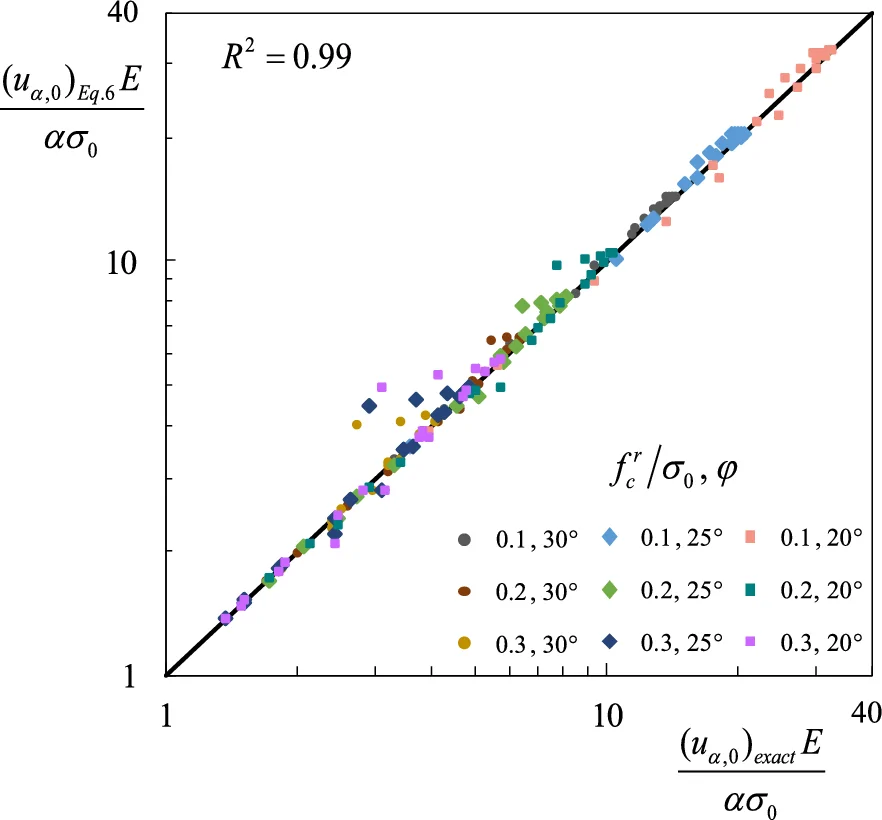
Quality of the analytical approximation for *λ* (Eq. 6). Comparison of the normalised tunnel convergences computed using the approximate *λ* versus the numerically derived *λ* for the reference circular tunnel case (*f*_*c*_ ^*r*^/*σ*_*0*_ = 0.1, 0.2 and 0.3, *φ* = 20º, 25º and 30º, *f*_*c*_ ^*p*^/*f*_*c*_ ^*r*^ = 1.5, 2, 3, 4 and 5, *γ* ^*pl*^_*95*_* Ε*/*σ*_*0*_ = 1, 4, 10 and 40 and *v* = 0.3).

The quality of this fit is illustrated by the red lines in Figs. 1, 2 and 3 and by Fig. 4, which compares the unsupported tunnel convergences calculated using *λ* after Eq. 6 with those obtained using the exact *λ*. The *R*^*2*^ of 0.99 indicates very good agreement between the regression approximation and the computed results across the studied parameter range. However, as seen in Figs. 1, 2 and 3, the fit accuracy decreases for high strength ratios, *f*_*c*_ ^*p*^/*f*_*c*_ ^*r*^ > 4, combined with low *γ* ^*pl*^_*95*_ *Ε*/*σ*_*0*_ < 3. In these specific ranges, the analytical approximation tends to underestimate *λ* (predicting a lower *f*_*c*_^*eq*^), which results in a conservative prediction of the tunnel convergence. However, this seemingly significant inaccuracy in *λ* does not result in a considerable deviation in the predicted displacements (see Fig. 4 which includes cases with *f*_*c*_ ^*p*^/*f*_*c*_ ^*r*^ = 4 and 5 and *γ* ^*pl*^_*95*_ *Ε*/*σ*_*0*_ = 1). Nevertheless, for high precision in these parameter ranges, direct use of the nomograms (black lines in Figs. 1, 2 and 3 is recommended.

## Validation for general cases

The performance of the proposed equivalent perfectly plastic model is evaluated for problem layouts that differ from the rotationally symmetric benchmark used for determining the equivalent strength. Finite element stress analyses were performed using Abaqus^9^, to simulate tunnel excavation under plane-strain conditions for various scenarios. The strain-softening constitutive model (Eq. 1) was implemented in Abaqus via a user-defined material subroutine (UMAT), which employs the stress integration algorithm of Clausen^8^, explicitly updating cohesion based on the accumulated deviatoric plastic strain.

### Numerical setup

The validation includes horseshoe-shaped tunnel cross-sections and anisotropic *in-situ* stress fields defined by *K*_*0*_. The following cases were examined:

- Horseshoe tunnel: (a) *K*_*0*_ = 0.6, (b) *K*_*0*_ = 1.3 and (c) *K*_*0*_ = 1.0
- Circular tunnel: (d) *K*_*0*_ = 0.6

The selected cases were chosen to examine the adequacy of the equivalent-strength approach for non-circular tunnel cross-sections and non-hydrostatic in-situ stress fields. For each layout, simulations were run using both the strain-softening model (Sect. 2) and the equivalent perfectly plastic model.

**Fig. 5:**
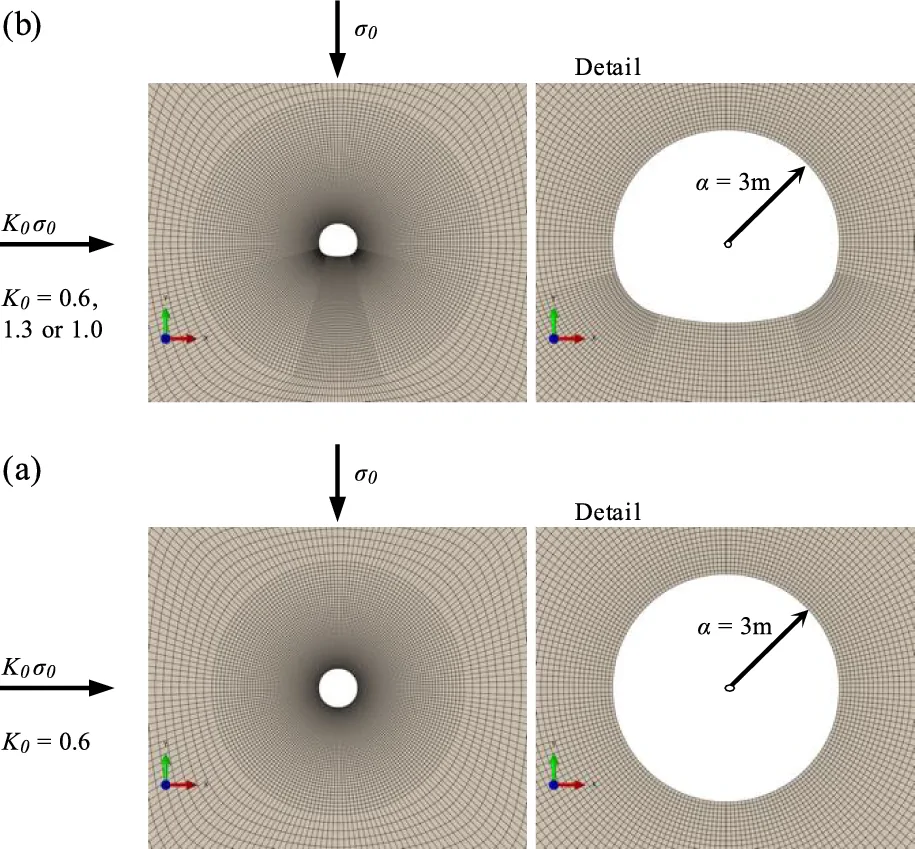
Part of the computational domain (finite element size at the excavation boundary: 100 × 100 mm).

Figure 5 illustrates part of the computational domain and spatial discretisation with 100 × 100 mm elements at the excavation boundary. The far field boundary is located 200 m away from the tunnel axis in both vertical and horizontal directions. To determine the convergence of an unsupported tunnel, the tractions at the excavation boundary are gradually reduced from their initial values (according to the *in-situ* stress field) to zero.

**Fig. 6:**
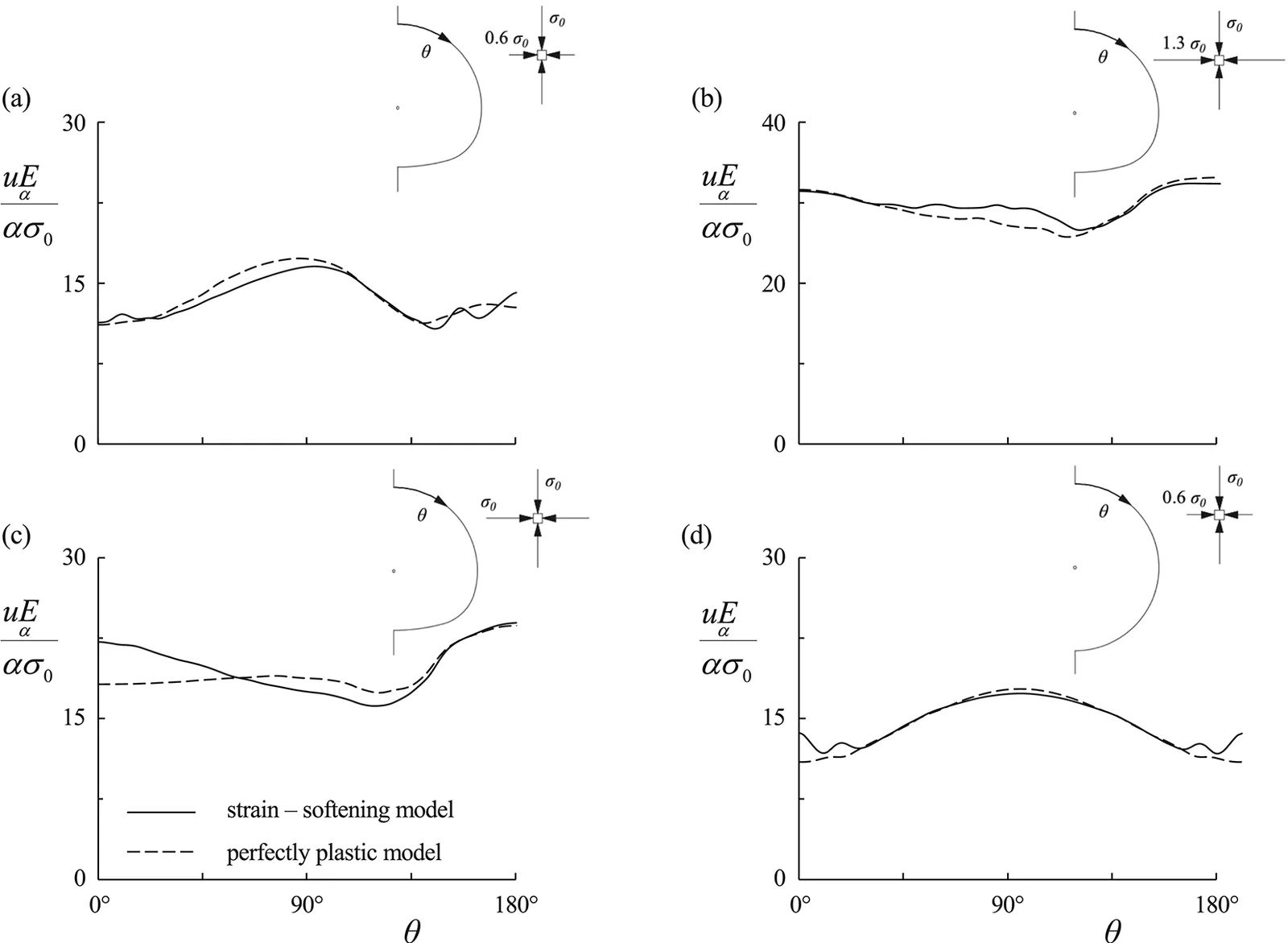
Comparison of normalised convergence computed numerically with the strain-softening model and the equivalent perfectly plastic model for different layouts (material parameters: *γ*^*pl*^_*95*_ *Ε*/*σ*_*0*_ = 10, *f*_*c*_ ^*r*^/*σ*_*0*_ = 0.1, *f*_*c*_ ^*p*^/*f*_*c*_ ^*r*^ = 1.5,* φ* = 25º, *ψ* = 5º and *v* = 0.3).

**Fig. 7:**
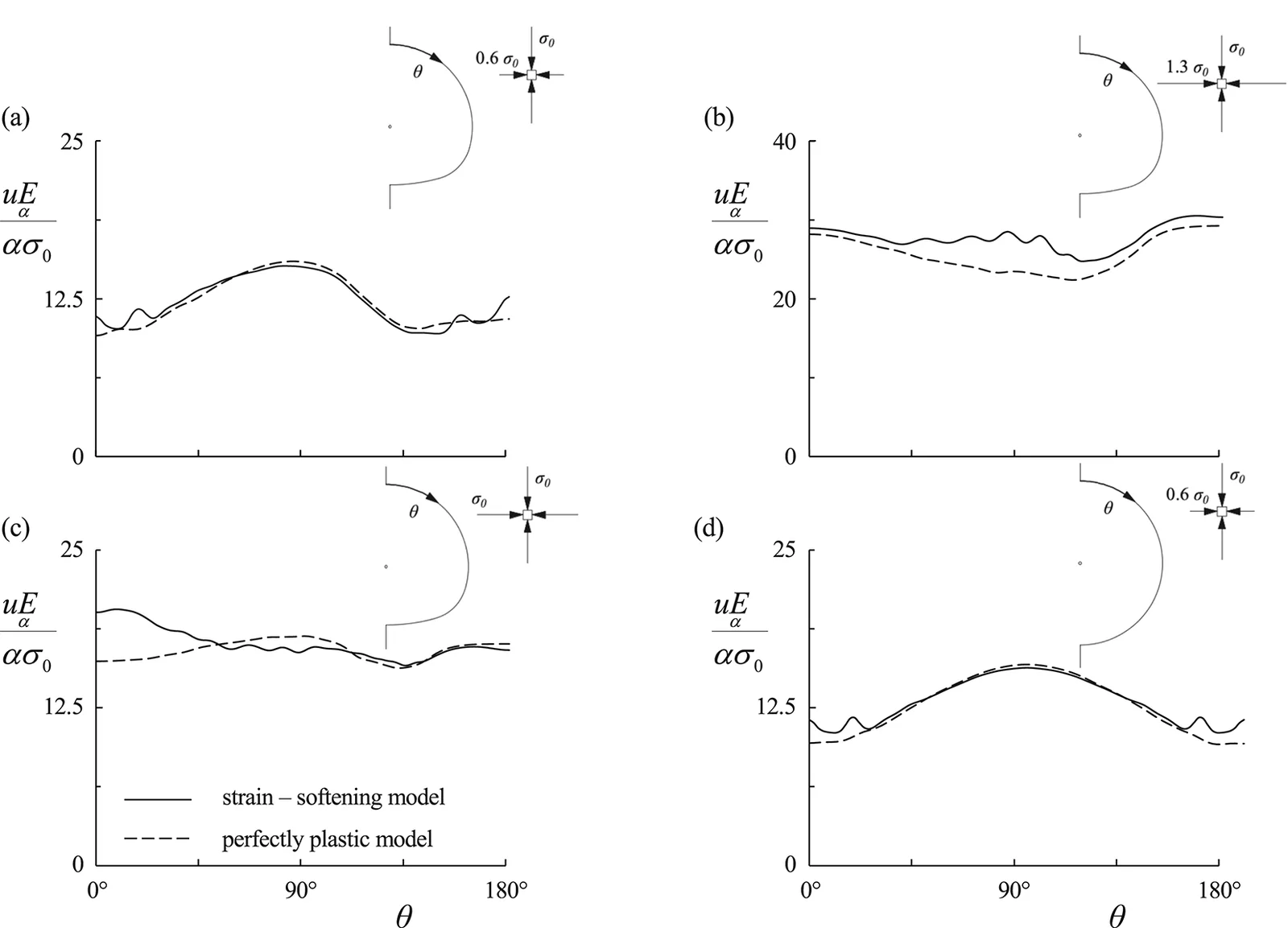
Comparison of normalised convergence computed numerically with the strain-softening model and the equivalent perfectly plastic model for different layouts (material parameters: *γ*^*pl*^_*95*_ *Ε*/*σ*_*0*_ = 30, *f*_*c*_ ^*r*^/*σ*_*0*_ = 0.1, *f*_*c*_ ^*p*^/*f*_*c*_ ^*r*^ = 1.5,* φ* = 25º, *ψ* = 5º and *v* = 0.3).

**Fig. 8:**
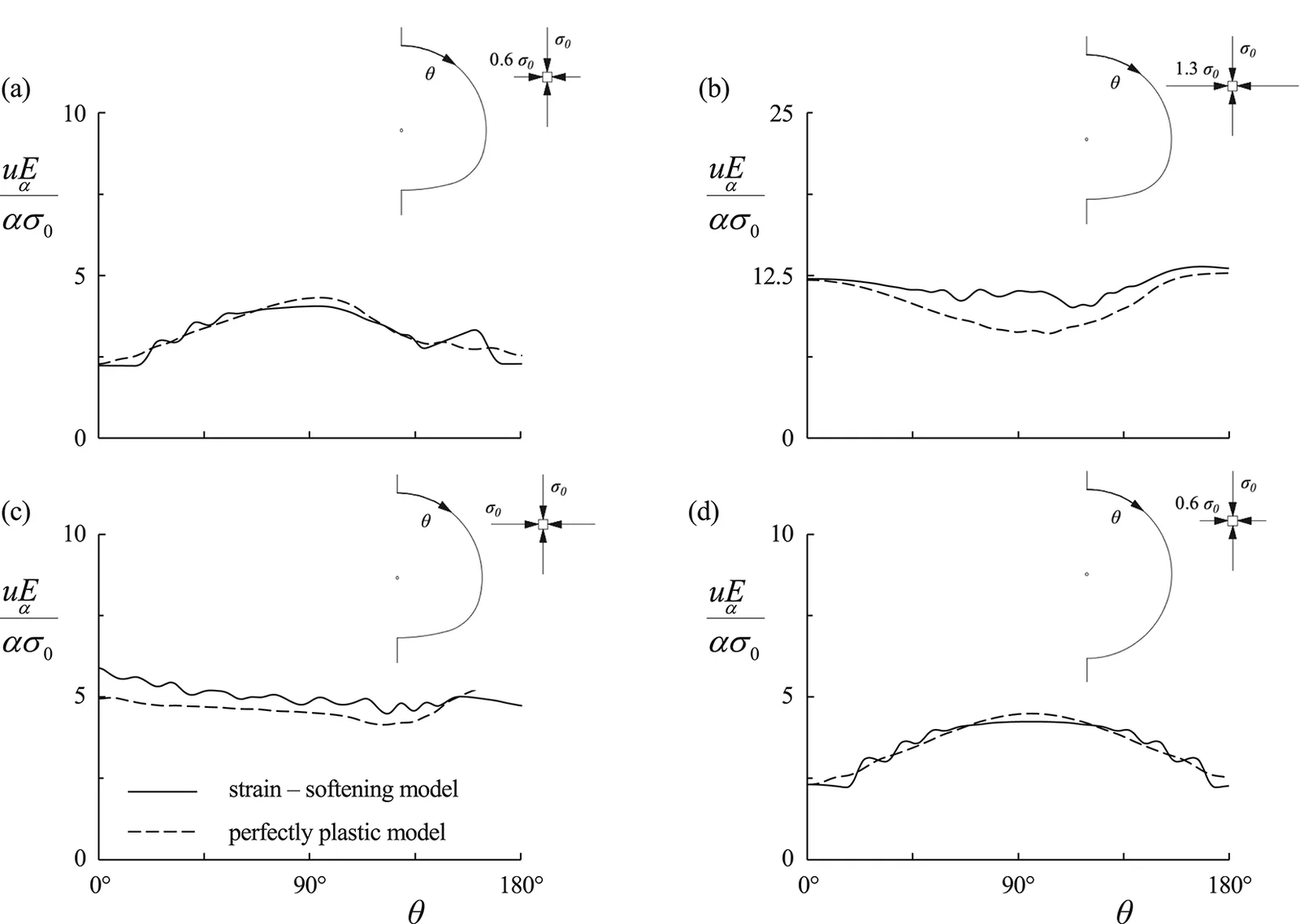
Comparison of normalised convergence computed numerically with the strain-softening model and the equivalent perfectly plastic model for different layouts (material parameters: *γ*^*pl*^_*95*_ *Ε*/*σ*_*0*_ = 10, *f*_*c*_ ^*r*^/*σ*_*0*_ = 0.2, *f*_*c*_ ^*p*^/*f*_*c*_ ^*r*^ = 1.5,* φ* = 25º, *ψ* = 5º and *v* = 0.3).

**Fig. 9:**
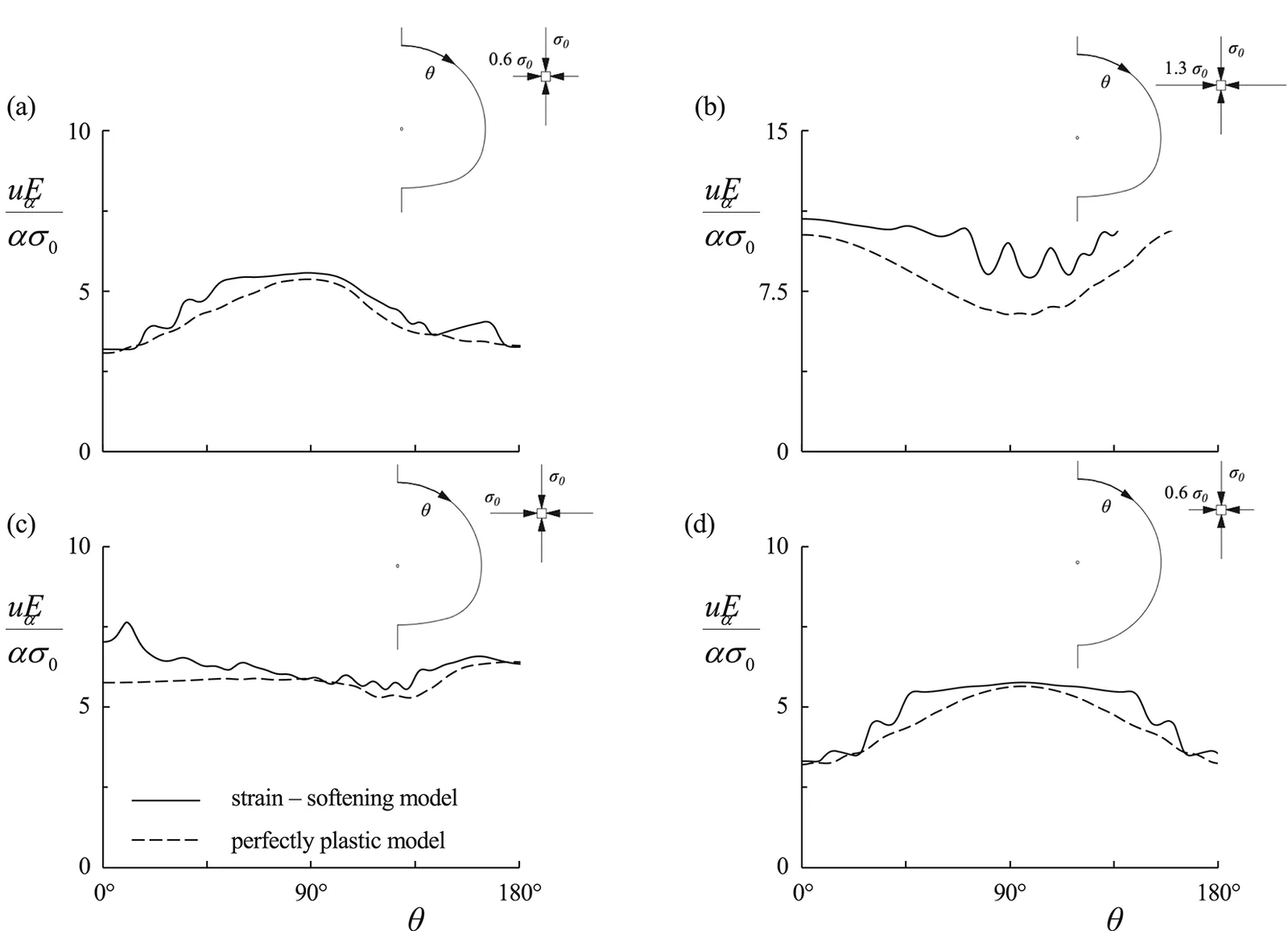
Comparison of normalised convergence computed numerically with the strain-softening model and the equivalent perfectly plastic model for different layouts (material parameters: *γ*^*pl*^_*95*_ *Ε*/*σ*_*0*_ = 30, *f*_*c*_ ^*r*^/*σ*_*0*_ = 0.2, *f*_*c*_ ^*p*^/*f*_*c*_ ^*r*^ = 1.5,* φ* = 25º, *ψ* = 5º and *v* = 0.3).

**Fig. 10:**
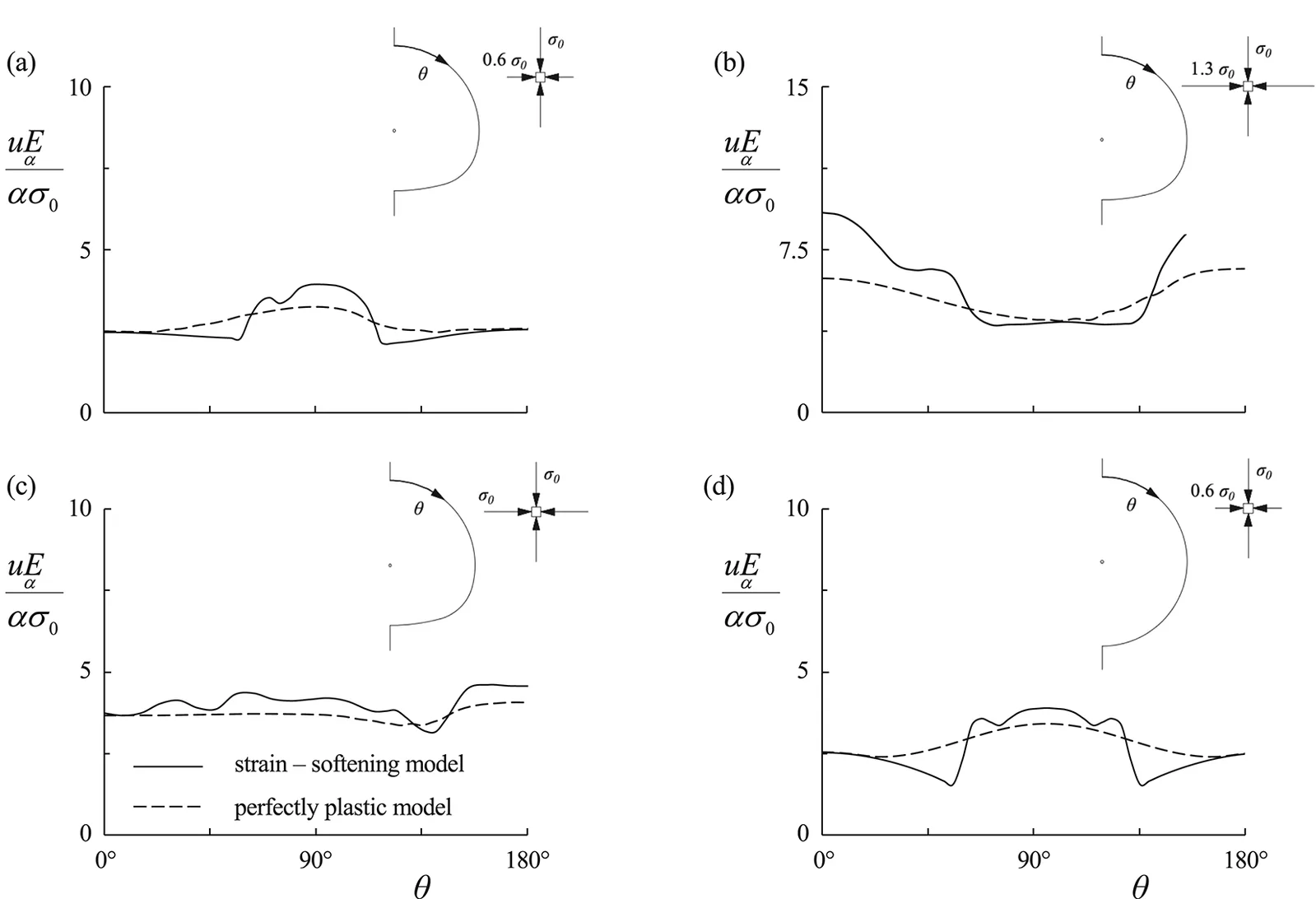
Comparison of normalised convergence computed numerically with the strain-softening model and the equivalent perfectly plastic model for different layouts (material parameters: *γ*^*pl*^_*95*_ *Ε*/*σ*_*0*_ = 10, *f*_*c*_ ^*r*^/*σ*_*0*_ = 0.2, *f*_*c*_ ^*p*^/*f*_*c*_ ^*r*^ = 3.5,* φ* = 25º, *ψ* = 5º and *v* = 0.3).

The meshes are generally fine, though not sufficient to fully resolve thin shear bands if strain localisation were to occur. The resulting numerical solution therefore corresponds to the non-localised response, consistently with the benchmark adopted for calibration. For the slightly softening case (*f*_*c*_ ^*p*^/*f*_*c*_ ^*r*^ = 1.5) examined in Figs. 6, 7, 8 and 9, the finite element size at the excavation boundary is 100 × 100 mm. For the severe softening case (*f*_*c*_ ^*p*^/*f*_*c*_ ^*r*^ = 3.5) examined in Fig. 10, the element size is 200 × 200 mm in cases (a) and (d), and 400 × 400 mm in cases (b) and (c). In all analyses, the element size increases linearly with distance from the tunnel axis.

The strength *f*_*c*_^*eq*^ of the equivalent perfectly plastic model was calculated using Eq. (4) with *λ* obtained from the nomograms (Figs. 1, 2 and 3). In the cases with *K*_*0*_ ≠ 1, *σ*_*0*_ denotes the vertical *in-situ* stress.

## Results

Figures 6, 7, 8, 9 and 10 compare the convergence pattern (i.e., displacement magnitude around the tunnel perimeter) predicted by the strain-softening model, serving as the reference non-localised numerical solution, and by the equivalent perfectly plastic model, for various material parameter sets and problem layouts.

The equivalent perfectly plastic model generally provides a good approximation of the convergence predicted by the strain-softening model. Both the magnitude and the pattern of displacements around the openings show reasonable agreement, despite *f*_*c*_^*eq*^ being derived from the simpler rotationally—symmetric case. The observed agreement across different geometries, stress conditions and softening characteristics supports the utility of the proposed equivalent-strength approach for practical estimation of non-localised tunnel convergence.

## Conclusions

This paper presented a practical method for approximating non-localised tunnel convergence in strain-softening ground using a computationally efficient perfectly plastic Mohr–Coulomb model. The appropriate value of the uniaxial compressive strength is determined such that the model reproduces the tunnel convergence predicted by Lee and Pietruszczak’s^15^ semi-analytical non-localised strain-softening solution for the rotationally symmetric problem of an unsupported circular tunnel. Dimensionless nomograms (Figs. 1, 2, and 3) and a closed-form analytical approximation (Eq. 6) were provided for determining *f*_*c*_^*eq*^ for a broad range of material parameters *f*_*c*_ ^*p*^/*f*_*c*_ ^*r*^, *f*_*c*_ ^*r*^/*σ*_*0*_, *γ*^*pl*^_*95*_ *Ε*/*σ*_*0*_,* φ*, *ψ* and *v*.

Validation through finite element analyses showed that the perfectly plastic model using the equivalent strength generally provides a good approximation of tunnel convergence magnitude and distribution predicted by the non-localised strain-softening solution, even for problem layouts involving horseshoe tunnel geometries and anisotropic *in-situ* stress fields. Similarly to Lee and Pietruszczak’s^15^ solution, which is used to determine the equivalent strength, and to other non-localised solutions from the literature, the method does not account for the local increase in deformations due to the formation of thin shear bands. However, it shares the advantages of these solutions in that it allows robust, computationally inexpensive and numerically stable preliminary estimations of squeezing deformations in problems without rotational symmetry. The proposed method therefore offers a practical means for estimating the non-localised response of strain-softening ground using constitutive models and analysis tools widely employed in engineering practice.

## Data Availability

The datasets generated and analysed during the current study are available from the corresponding author on reasonable request.

## Latin symbols

*a*: Tunnel radius
$a_{k}$: Curve fitting parameters (k = 1:4)
*E*: Young’s modulus
$f_{c}$: Uniaxial compressive strength
$$f_{c}^{{eq}}$$: Equivalent strength
$$f_{c}^{p}$$: Peak strength
$$f_{c}^{r}$$: Residual strength
$K_{o}$: Ratio of horizontal to vertical in-situ stress
$u_{{a,o}}$: Unsupported tunnel convergence

## Greek symbols

$\gamma ^{{pl}}$: Deviatoric plastic strain
$\gamma _{{95}}^{{pl}}$: Deviatoric plastic strain causing 95% of the total strength loss
*λ*: Softening factor
*ν*: Poisson’s ratio
$\sigma _{0}$: In-situ stress
*φ*: Friction angle
*ψ*: Dilatancy angle

## References

[1] Alonso, E., Alejano, L. R., Varas, F., Fdez-Manin, G. & Carranza-Torres, C. Ground response curves for rock masses exhibiting strain-softening behaviour. *Int. J. Numer. Anal. Methods Geomech.* **27**(13), 1153–1185 (2003).

[2] Anagnostou, G. & Kovári, K. Significant parameters in elasto-plastic analysis of underground openings. *Journal of Geotechnical Engineering* **119**(3), 401–419 (1993).

[3] Anthi, M., Pferdekämper, T. & Anagnostou, G. Analytical solutions for cavity contraction in strain-softening materials with linear or exponential strength decay. *Sci. Rep.* **14**, 15040. 10.1038/s41598-024-65186-y (2024). 38951535 PMC11217471

[4] Cantieni, L. & Anagnostou, G. The interaction between yielding supports and squeezing ground. *Tunn. Undergr. Space Technol.* **24**, 309–322 (2009).

[5] Cantieni, L., Nordas, A., Morosoli, D., Anagnostou, G. (2023): On the short-term response of Opalinus Clay to tunnelling. 15th ISRM Congress 2023 & 72nd Geomechanics Colloquium, 373–378, Schubert & Kluckner (eds.) © ÖGG

[6] Carranza-Torres, C. Self-similarity analysis of the elastoplastic response of underground openings in rock and effects of practical variables. *Ph.D. Thesis,* University of Minnesota (1998).

[7] Chen, W., Zhang, D., Fang, Q., Chen, X. & Xu, T. A new numerical finite strain procedure for a circular tunnel excavated in strain-softening rock masses and its engineering application. Appl. Sci. **12**(5), 2706 (2022).

[8] Clausen J. Efficient non-linear finite element implementation of elasto-plasticity for geotechnical problems. Ph.D. thesis, Esbjerg Institute of Technology, Aalborg University (2007)

[9] Dassault Systèmes. *ABAQUS 2019 Theory Manual* (Dassault Systèmes Simulia Corp., 2019).

[10] Einav, I. & Randolph, M. F. Combining upper bound and strain path methods for evaluating penetration resistance. *Int. J. Numer. Methods Eng.* **63**, 1991–2016 (2005).

[11] Gonzalez-Cao, J., Alejano, L. R., Alonso, E. & Bastante, F. G. Convergence-confinement curve analysis of excavation stress and strain resulting from blast-induced damage. *Tunn. Undergr. Space Technol.* **73**, 162–169 (2018).

[12] Guan, K. et al. A finite strain numerical procedure for a circular tunnel in strain-softening rock mass with large deformation. *Int. J. Rock Mech. Min. Sci.* **112**, 266–280 (2018).

[13] Hoek, E. & Brown, E. T. Practical estimates of rock mass strength. *Int. J. Rock Mech. Min. Sci.* **34**(8), 1165–1186 (1997).

[14] Hoek, E., Marinos, P. 2000. Predicting tunnel squeezing problems in weak heterogeneous rock masses.*Tunnels and Tunnelling International*, 32 (11 and 12) Part 1 – November 2000, Part 2 – December 2000, p. 45–51 and p. 34–36

[15] Lee, Y. & Pietruszczak, S. A new numerical procedure for elasto-plastic analysis of a circular opening excavated in a strain-softening rock mass. *Tunn. Undergr. Space Technol.* **23**, 588–599 (2008).

[16] Leone, T., Nordas, A. & Anagnostou, G. An estimation equation for the TBM thrust force in creeping rock. *Comput. Geotech.* **165**, 105802 (2024).

[17] Li, J., Shen, C., He, X., Zheng, X. & Yuan, J. Numerical solution for circular tunnel excavated in strain-softening rock masses considering damaged zone. *Sci. Rep.* **12**(1), 4465 (2022). 35296746 10.1038/s41598-022-08531-3 PMC8927610

[18] Martin, C. D. & Chandler, N. A. The progressive fracture of Lac du Bonnet granite. *Int. J. Rock Mech. Min. Sci. Geomech. Abstr.* **31**(6), 643–659 (1994).

[19] Mezger, F., Ramoni, M., Anagnostou, G., Dimitrakopoulos, A. & Meystre, N. Evaluation of higher capacity segmental lining systems when tunnelling in squeezing rock. *Tunn. Undergr. Space Technol.* **65**, 200–214 (2017).

[20] Mezger, F., Ramoni, M. & Anagnostou, G. Options for deformable segmental lining systems for tunnelling in squeezing rock. *Tunn. Undergr. Space Technol.* **76**, 64–75 (2018).

[21] Nordas, A., Natale, M., Leone, T. & Anagnostou, G. Thrust force requirements in fault zones with squeezing ground. *Comput. Geotech.* 10.1016/j.compgeo.2023.105479 (2023a).

[22] Nordas, A., Natale, M., Cantieni, L., Anagnostou, G. (2023b): Study on TBM jamming hazard in Opalinus clay. Expanding Underground. Knowledge and Passion to Make a Positive Impact on the World – Anagnostou, Benardos & Marinos (Eds). Proc. of the ITA-AITES World Tunnel Congress 2023 (WTC 2023), 12–18 May 2023, Athens, Greece, 2291–2298. 10.1201/9781003348030-258

[23] Nordas, A. N., Cantieni, L. & Anagnostou, G. An analytical solution for the undrained ground response to tunnelling considering the excavation-induced desaturation. *J. Rock Mech. Geotech. Eng.* 10.1016/j.jrmge.2024.04.020 (2024).

[24] Panet, M. Analysis of convergence behind the face of a tunnel. *Tunn. Undergr. Space Technol.* **10**(2), 153–161 (1995).

[25] Ramoni, M. & Anagnostou, G. Thrust force requirements for TBMs in squeezing ground. *Tunn. Undergr. Space Technol.* **25**, 433–455 (2010).

[26] Ramoni, M., Lavdas, N. & Anagnostou, G. Squeezing loading of segmental linings and the effect of backfilling. *Tunn. Undergr. Space Technol.* **26**, 692–717 (2011).

[27] Varas, F., Alonso, E., Alejano, L. R. & Manín, G. F. Study of bifurcation in the problem of unloading a circular excavation in a strain-softening material. *Tunn. Undergr. Space Technol.* **20**(4), 311–322 (2005).

[28] Vermeer, P. A. & de Borst, R. Non-associated plasticity forsoils, concrete and rock. *Heron* **29**(3), 1–64 (1984).

[29] Vrakas, A., Dong, W. & Anagnostou, G. Elastic deformation modulus for estimating convergence when tunnelling through squeezing ground. *Géotechnique* 10.1680/jgeot.17.P.008 (2017).

[30] Zhang, Q., Wang, H. Y., Jiang, Y. J., Lu, M. M. & Jiang, B. S. A numerical large strain solution for circular tunnels excavated in strain-softening rock masses. *Comput. Geotech.* **114**, 103142 (2019).

[31] Zhang, Q., Wang, X. F., Jiang, B. S., Liu, R. C. & Li, G. M. A finite strain solution for strain-softening rock mass around circular roadways. *Tunn. Undergr. Space Technol.* **111**, 103873 (2021).

[32] Zhang, Q., Zhang, C., Jiang, B., Li, N. & Wang, Y. Elastoplastic coupling solution of circular openings in strain-softening rock mass considering pressure-dependent effect. *Int. J. Geomech.* **18**(1), 04017132 (2018).

[33] Zou, J. F., Li, C. & Wang, F. A new procedure for ground response curve (GRC) in strain-softening surrounding rock. *Comput. Geotech.* **89**, 81–91 (2017).

